# Measuring Cognitive Load Using In-Game Metrics of a Serious Simulation Game

**DOI:** 10.3389/fpsyg.2021.572437

**Published:** 2021-03-24

**Authors:** Natalia Sevcenko, Manuel Ninaus, Franz Wortha, Korbinian Moeller, Peter Gerjets

**Affiliations:** ^1^Daimler Trucks AG, Stuttgart, Germany; ^2^Department of Psychology, Faculty of Science, Eberhard Karls University, Tübingen, Germany; ^3^Leibniz-Institut für Wissensmedien, Tübingen, Germany; ^4^LEAD Graduate School and Research Network, Eberhard Karls University, Tübingen, Germany; ^5^Centre for Mathematical Cognition, School of Science, Loughborough University, Loughborough, United Kingdom

**Keywords:** cognitive load, in-game metric, adaptivity, serious games, simulation

## Abstract

Serious games have become an important tool to train individuals in a range of different skills. Importantly, serious games or gamified scenarios allow for simulating realistic time-critical situations to train and also assess individual performance. In this context, determining the user’s cognitive load during (game-based) training seems crucial for predicting performance and potential adaptation of the training environment to improve training effectiveness. Therefore, it is important to identify in-game metrics sensitive to users’ cognitive load. According to Barrouillets’ time-based resource-sharing model, particularly relevant for measuring cognitive load in time-critical situations, cognitive load does not depend solely on the complexity of actions but also on temporal aspects of a given task. In this study, we applied this idea to the context of a serious game by proposing in-game metrics for workload prediction that reflect a relation between the time during which participants’ attention is captured and the total time available for the task at hand. We used an emergency simulation serious game requiring management of time-critical situations. Forty-seven participants completed the emergency simulation and rated their workload using the NASA-TLX questionnaire. Results indicated that the proposed in-game metrics yielded significant associations both with subjective workload measures as well as with gaming performance. Moreover, we observed that a prediction model based solely on data from the first minutes of the gameplay predicted overall gaming performance with a classification accuracy significantly above chance level and not significantly different from a model based on subjective workload ratings. These results imply that in-game metrics may qualify for a real-time adaptation of a game-based learning environment.

## Introduction

Serious games have become an important tool for educating and training people in a variety of different skills, ranging from military purposes to education and health care (for an overview see: Susi et al., 2007; Boyle et al., 2016); Unlike traditional analog learning, which cannot be automatically adapted to individual needs, serious games and simulations can be programmed to create targeted learning programs. While digital training in areas such as maths, language learning, exercise, or healthy eating can easily be replaced by analog setups, a range of situations such as aircraft crashes, surgical operations, or – more generally – time-critical emergency situations, can hardly be trained in real-life situations, it may benefit considerably from simulations and/or serious games. The most pronounced advantage of such digital training consists not only of the potential to simulate dangerous and time-critical situations, hard to recreate in analog surroundings but also of the fact that any digital training system also allows for the collection of individual in-game metrics (e.g., performance progression or computer mouse/keyboard usage) upon which learning analytics can be applied (Freire et al., 2016). Measures such as memory and learning outcomes may directly be used for an adjustment of difficulty levels of the learning environment. However, these outcome measures are usually only available after a particular task has been completed. In contrast, estimations of players’ cognitive or emotional states based on in-game metrics (Nebel and Ninaus, 2019), might be used to adapt systems to increase training effectiveness, performance, and motivation. Among different affective and cognitive components, cognitive load seems to be particularly interesting as it is considered to reflect the degree to which available cognitive resources are engaged in the task at hand (Babiloni, 2019). As Gerjets et al. (2014) pointed out, the actual level of cognitive load is relevant in a variety of realistic settings, such as adaptive learning environments, where optimal learning content is characterized by an intermediate level of cognitive load. The researcher showed that the learners’ cognitive load while solving complex realistic tasks can be classified by analyzing electroencephalography (EEG) data using machine learning algorithms. Moreover, previous results indicated that adaptations based on measured cognitive load can lead to significant learning improvements comparable to effects of failure-based adaptations, even when a generalized prediction model without user-specific calibration is used (Walter et al., 2017).

In the current study, we used a serious simulation game for training emergency personnel with the aim to assess participants’ cognitive load by in-game metrics using a theory-driven approach. Below we provide a brief overview of cognitive load and its measurement methods. This is followed by a more detailed description of the time-based resource-shared (TBRS) model of Barrouillet et al. (2004), which provides the theoretical foundation for our approach on in-game metrics measuring cognitive load before we describe the details of the current study and hypotheses.

### Cognitive Load and Adaptation to Cognitive Load

The concept of cognitive load goes back to the finding that working memory capacity is limited to approx. Seven chunks of information (Miller, 1956), and thus cognitive resources, in general, are limited. According to the definition of Paas and Van Merriënboer (1994), cognitive load is a multidimensional construct and represents demands that a particular task imposes on the cognitive system. While this definition offers a good initial idea of the construct, the theoretical details of how cognitive load should be precisely conceptualized are still under discussion. Thus, even though the research on cognitive load has a long history (Linton et al., 1978; Welford, 1978; Sheridan and Simpson, 1979; Eggemeier et al., 1985; Meshkati, 1988; Sweller et al., 1998; Barrouillet et al., 2004) it’s still a scientifically vibrant field of interest given its crucial importance for everyday life. As noted by Babiloni (2019) in his recent review on the topic, cognitive load can be characterized by a complex interplay between different task demands and a variety of mental processes such as alertness, vigilance, fatigue, etc., and thus represents a result of a complex interaction of different aspects. That is, cognitive load is a dynamic variable that may change rapidly during task processing. Nevertheless, three general assumptions regarding the construct of cognitive load can be derived from the literature (cf. Babiloni, 2019). First, human cognitive and attentional resources are limited. Second, different tasks can require different cognitive resources to varying degrees. And third, different individuals may experience different levels of cognitive load when conducting a task even when achieving the same performance level on it.

Ample evidence emphasizes the importance of cognitive load in our everyday life. For instance, cognitive load plays a crucial role in performing everyday activities such as learning/education (Ruiz et al., 2010), car driving (Kohlmorgen et al., 2007; Hancock et al., 2012), rail industry (Fan and Smith, 2017), air force (Hancock, 1989), office work (Smith-Jackson and Klein, 2009), and medicine (Yurko et al., 2010). Thus, accurately measuring cognitive load seems of considerable importance for a better understanding of the fluctuations in human performance.

According to an influential theoretical account, the relationship between cognitive load and performance is non-linear and can be described following an “inverted-U” shaped function (Veltman and Jansen, 2005; Babiloni, 2019), see also Yerkes and Dodson (1908). Importantly, the general idea of this “inverted-U” shaped relationship is also closely related to the concept of “flow” proposed by Csikszentmihalyi (1987). Flow is described as a positive emotional and cognitive state (Kiili et al., 2018) of optimal concentration and absorption. The state of flow is achieved when there is a good balance between the demands of a given task and the perceived skills and resources of an individual to solve the task. That is, a given task should not be too difficult (i.e., cognitive overload) or too easy (i.e., cognitive underload and boredom) to elicit a flow state allowing for optimal performance. Consequently, optimal learning content should be moderately challenging but should neither induce cognitive over- nor underload. The very same consideration is also reflected in classical theories of instructional design. So moderately challenging optimal training state corresponds to the “zone of proximal development” (cf. Vygotsky, 1980) and “amount of invested mental effort” (cf. Salomon, 1984).

Empirical evidence substantiated this theorized relationship between cognitive load and performance. For instance, Cummings and Nehme (2009) evaluated the relationship between cognitive load and performance of operators supervising multiple unmanned vehicles during a simulation of a military mission. In a series of two experiments, they showed that the addition of non-linear parabolic components into their performance prediction model improved its predictive power significantly. This demonstrated the non-linear character of this relationship and indicates that individuals perform best at medium levels of cognitive load (e.g., Anderson, 1994; Watters et al., 1997; Montani et al., 2020). These results generalize to educational environments as well as serious games and reflecting that to achieve the best learning outcomes learners should be kept in an intermediate range of cognitive load where they are not bored (Pekrun et al., 2010) but also not overstrained (Niederhauser et al., 2000; Chang et al., 2018; Geng and Yamada, 2020).

In this way, it becomes clear that an ideal learning environment should not only be tailored to the specific needs of the learners (Gerjets and Hesse, 2004; Richards et al., 2007), for instance distinguishing between different expertise levels (cf. “expertise reversal effect” by Kalyuga, 2007). But also needs to consider that cognitive load is a dynamic variable that depends on different cognitive processes and may change during task accomplishment. Therefore, in order to keep learners within an optimal intermediate range of cognitive load, such systems should be able to identify undesirable states of under- and overload in real-time and adapt an ongoing task accordingly. In this way, performance and learning outcomes might be optimized.

Empirical evidence suggests that such online adaptation is indeed practicable (Gerjets et al., 2014; Appel et al., 2019) and can improve performance. For instance, Kohlmorgen et al. (2007) examined whether an adaptive reduction of cognitive load would lead to improved performance in a real-world driving task. Using EEG they were able to detect drivers’ cognitive overload and to adapt to it accordingly by making the task easier. In turn, this led to improved driving performance. Similarly, Yuksel et al. (2016) reported better performance as a result of adapting task difficulty to cognitive load. They used near-infrared spectroscopy (NIRS) to detect states of cognitive underload in pianists during a musical learning task and increased difficulty of the respective lessons accordingly. Moreover, Walter et al. (2017) developed a learning environment that adapted task difficulty based on EEG recordings reflecting the cognitive load of learners. Optimal cognitive load was deduced from EEG data and was not individually calibrated. Nonetheless, this system led to learning outcomes similar to that observed for error-based adaptation.

These examples indicate the growing popularity of this approach and its importance for future studies. However, they also point to the diversity of measurement techniques in this field. The following section introduces and classifies different ways of measuring cognitive load.

#### Measurement of Cognitive Load

Cognitive load assessment techniques that might be used to guide adaptations to cognitive load should be able to respond sensitively to variations in cognitive demands of the task at hand or interaction with learning systems without causing external disturbances to performance on the primary task (Orru and Longo, 2018). The literature distinguishes between four main categories of cognitive load measurement techniques: subjective measures, performance measures, behavioral measures, and physiological measures (Johannsen, 1979; Eggemeier et al., 1991; Scerbo, 1996; Brünken et al., 2010).

##### Subjective Measures

Subjective measurements are based on the observation that people are able to interpret and adequately describe their experienced cognitive load during a particular task (Gopher and Braune, 1984). These self-reported descriptions are collected using questionnaires such as SWAT (Reid and Nygren, 1988) and NASA-TLX (Hart and Staveland, 1988), which require participants to rate their experiences using predefined scales immediately after completing a specific task. Subjective measures are easy to collect, they are inexpensive and they usually provide consistent results (O’Donnell and Eggemeier, 1986). Therefore, these measures are widely accepted and have been thoroughly evaluated. Despite their advantages, subjective measurements have also a number of limitations. The main issue is that responding to a questionnaire interrupts task execution and thus can only be carried out after the task has already been completed, which has some potentially confounding consequences. Firstly, a retrospective view of an experienced cognitive load may be distorted by fading memory. Secondly, experienced failures (or successes) can bias the *post hoc* perception of cognitive load (Hancock, 1989). Thirdly, only a rough summary of the experience can be grasped in this way, which is not capable of tracking fine variations of cognitive load over time. And finally, self-reported measurements are only able to reflect conscious aspects of the cognitive load experienced during task accomplishment.

##### Performance Measures

Performance-based approaches evaluate variations in human performance. Based on empirical evidence, performance should decrease in case of cognitive overload (Yerkes and Dodson, 1908; Veltman and Jansen, 2005; Babiloni, 2019). Accordingly, a drop of performance may help to detect cognitive overload. As a main objective of cognitive load measurement is the prediction of task performance, this cluster of measurement techniques appears intuitively to be the most obvious and direct to apply. Unfortunately, it cannot be determined whether observed variations in performance have actually occurred due to changes in cognitive load or due to other relevant factors such as arousal or motivation (Brünken et al., 2010). Therefore, these measures yield no independent assessments of cognitive load for performance prediction. Moreover, in many cases it is not possible to obtain performance data during actual task completion, so that performance-based measurements can very often only be calculated and analyzed post-factum, rendering them useless for prediction or adaptation.

##### Behavioral Measures

Behavioral measures rely on the analysis of differences in interaction behavior during task processing, such as speech and voice patterns (Berthold and Jameson, 1999; Ruiz et al., 2010; Magnusdottir et al., 2017) or differences in the usage of input modalities such as keyboard or mouse (Ikehara and Crosby, 2005; Lim et al., 2014). These measures are usually unobtrusive and do not distract participants from the task at hand. Moreover, they do not require additional equipment and are usually inexpensive. Behavioral measures potentially allow for a continuous online measurement of cognitive states during task execution. However, identifying in the data related to cognitive load behavioral patterns is by no means a trivial endeavor, as these behavioral patterns might also be influenced by other factors such as emotions or stress.

##### Physiological Measures

Physiological measures of cognitive load rely on detecting physiological changes associated with cognitive states (Johannsen, 1979). Depending on the type of signal to be recorded, they can be more or less obtrusive. While sensors for electrodermal activity (EDA) or heart rate variability (HRV) can be rather discreet, EEG or functional magnetic resonance imaging (fMRI) are less practical or even impracticable in real-life situations because of their complexity, immobility, and obtrusiveness (for an overview see Ninaus et al., 2013). One major advantage of physiological measures is that they allow for continuous online recording. However, physiological measures require special equipment, cause additional costs, and the detection of cognitive states based on physiological signals is also not a trivial task (Gerjets et al., 2014; Appel et al., 2019). Because physiological processes are not only driven by cognitive states but can also be influenced by a variety of other factors, such as motor actions or emotions, it is not always unambiguously clear whether a change in a physiological signal was actually caused by the targeted cognitive state (Kramer, 1991). Moreover, physiological signals often require user-specific calibrations due to the signals’ high inter-subject variability.

##### Conclusion

While there seem to be numerous methods for measuring cognitive load, a perfect single assessment approach capable of capturing all relevant facets of cognitive load, preferably in real-time, simply does not exist. In recent years, a trend towards the development of complex multimodal measurement systems to capture cognitive load can be observed (Ikehara and Crosby, 2005; Herff et al., 2014; Zhou et al., 2020). However, due to their inherent complexity, multimodal approaches seem to be primarily useful for extensive online data acquisition in the laboratory. In real-world scenarios outside the laboratory, such as gameplay, it seems reasonable to focus on metrics that on the one hand reflect users’ behavior and performance and on the other hand can be easily collected during gameplay without requiring additional equipment. In view of future developments, such simple but reliable metrics might also become part of more complex monitoring systems. However, as argued above, changes in users’ behavior and performance do not necessarily directly reflect changes in cognitive load, so that a solid theoretical framework for the development of such metrics will be needed. In this paper, we will rely on the TBRS model described below to provide a suitable theoretical basis for assessing cognitive load based on behavioral and performance measures in time-critical multitasking environments requiring simultaneous execution of several tasks under severe time constraints.

### The Time-Based Resource-Sharing Model

Time-based resource-shared (for a comprehensive overview of the model and its development history see Barrouillet et al., 2004; Barrouillet and Camos, 2015) describes working memory as the core system of cognition dedicated to the processing and storage of information, whereby both storage and processing components of working memory are required for the execution of a cognitive task. This idea can be illustrated by a simple example. Considering an arithmetic task, such as two-digit multiplication, the processing component would be occupied with arithmetic operations, while the storage component would be needed to memorize intermediate results. Similarly, in a reading task, one needs to remember the context of what is currently being read as well as to decode a sequence of words to understand the meaning of a new sentence.

From these examples, it seems intuitively clear that processing components of working memory requires attention, but at the same time one must also somehow “refresh” the intermediate results of processing by means of intentionally thinking about them. That means that attention must be shared between both components of working memory. This idea is responsible for the second part of the models’ name as TBRS assumes that attention is a limited *resource* that must be *shared* in a way that *only one* central process such as storage or processing of information can be performed at a time. As soon as attention is directed to the processing component (e.g., to an arithmetic operation), the stored information (e.g., an intermediate result from a previous calculation step) will begin to fade from memory. This so-called decay of memory traces is progressing in the time during which attention is captured due to the ongoing calculation process. However, TBRS postulates that simultaneous task execution can be mimicked by rapid switches of attention between to-be-performed subtasks – potentially interrupting the processing component of the current task (i.e., one may briefly interrupt a simple arithmetic operation to remind oneself of the intermediate result). This leads to a complex and time-critical interplay between executed processing and storage activities yielding that attention sharing happens in a *time-based* manner, which explains the full name of the TBRS model: TBRS model.

Coming back to the example of a two-digit multiplication, what if a subject is perhaps very young and not very skilled in this type of task so that the arithmetic operation captures all of his attention without providing the storage process with any chance of refreshing intermediate results? Probably, after some time these results cannot be retrieved from memory anymore so that further calculations would be rendered impossible, yielding a drop in performance. In the contrary, for a very skilled subject, the arithmetic operations might be carried out in a more automated way requiring less attention, so that it would not be difficult to “refresh” intermediate results and show optimal performance. Taking these considerations into account, TBRS predicts that cognitive load and thus performance will depend on the proportion of time during which attention is captured in such a way that the storage of information is disturbed.

As Barrouillet et al. (2004) emphasizes, it is unfortunately not trivial to determine the exact time during which attention is captured by processing demands. Moreover, as the model was developed and evaluated mainly for working-memory span tasks (Daneman and Carpenter, 1980; Case et al., 1982), it’s further evaluation and extension to other executive functions required the design of specific experimental paradigms, allowing for defining certain retention and storage intervals at a predefined pace (Lépine et al., 2005; Barrouillet et al., 2007; Camos et al., 2007; Liefooghe et al., 2008; Portrat, 2008).

As such fine-tuned and hard-paced settings are hardly present in everyday life, two questions arise: Can the model also be applied to more realistic setups, and if so, how should such setups look like. A real-life situation that comes closest to a hard-paced working-memory span task may be a computer-based test with restricted execution time for particular subtasks. Another situation with inherited pacing could be a time-critical management situation in which the pace is determined indirectly due to the reaction and execution time of available resources. However, as in both described situations, separation into subtasks would be more difficult than in a working memory span task, it remains unclear how the model and its prediction of the resulting cognitive load can be used in more general situations, such as serious games, where the pace is only indirectly determined while time pressure is still relevant. In this study, we aimed to address this question by proposing an in-game metric for measuring cognitive load based on the theoretical framework of TBRS.

### The Present Study

Determining cognitive load during a serious game might be crucial for performance predictions as well as for providing adaptations to improve learning outcomes. In this study, we aimed at evaluating a practicable and parsimonious solution for cognitive load detection in serious games based on TBRS with regard to its reliability and potential suitability for online assessments and evaluations. To validate our approach we used commonly applied subjective reports of cognitive load as assessed by the NASA-TLX (Hart and Staveland, 1988). We focused on the use of in-game metrics based on users’ behavior and performance as sources of information because these measures can be easily collected during gaming without extra equipment and provide relevant empirical evidence in terms of the TBRS model Barrouillet et al. (2004). The validity of the proposed metrics for predicting cognitive load was evaluated in terms of their relation to the cognitive load as reported in the NASA-TLX and to the overall gaming performance. To implement sufficient variance in cognitive load we used an adaptation of a complex serious game simulating an emergency situation with different scenarios and levels of difficulty.

In particular, we pursued the following hypotheses. First, proposed measures of cognitive load based on in-game metrics as well as subjective self-report should validly reflect differences between various scenarios and levels of difficulty as a manipulation check. We expected that cognitive load should be higher in more difficult scenarios and levels as indicated by both in-game metrics as well as subjective ratings. Second, on an individual level, we expected that cognitive load as indicated by the in-game metrics used should be associated significantly with participants’ subjective rating of their cognitive load as measured by the NASA-TLX, as well as with their overall gaming performance. Third, we hypothesized that the in-game metrics developed should allow for the prediction of overall gaming performance comparably well as subjective ratings provided by the NASA-TLX.

## Methods

This study focused on measuring cognitive load with behavioral in-game metrics. It was carried out as part of a larger project that included several other physiological measures such as functional NIRS (fNIRS), cardiac measurements, galvanic skin response, and eye-tracking (cf. Appel et al., 2019). As the aim of the current study was the evaluation of a simple and practicable parsimonious solution for cognitive load detection in serious games, the current analyses solely focused on behavioral and performance measures.

### Participants

Forty-seven volunteers (33 females, 14 males) aged between 15 and 49 years (*M* = 24.6; *SD* = 6.4) participated in the study with most of them being students (95.7%). Informed consent was obtained from all participants or their parents when under the age of 18 (one participant). All participants were right-handed, fluent in German, recruited *via* an online database, and compensated with 8 EUR for completing the study. The study was approved by the local ethic committee and a written informed consent was obtained. Participants reported no neurological, psychiatric, cardiovascular disorders, and did not take any psychotropic medications.

### Task

Participants played a customized version of the serious game *Emergency* (Promotion Software GmbH, 1999), which provides simulations of different emergency situations. The game comprised different scenarios with three levels of difficulty each. During gaming, participants’ task was to coordinate six types of emergency personnel, such as paramedics, emergency doctors, firefighters, ambulances, as well as fire- and ladder trucks, to rescue victims and extinguish fires.

**Figure 1:**
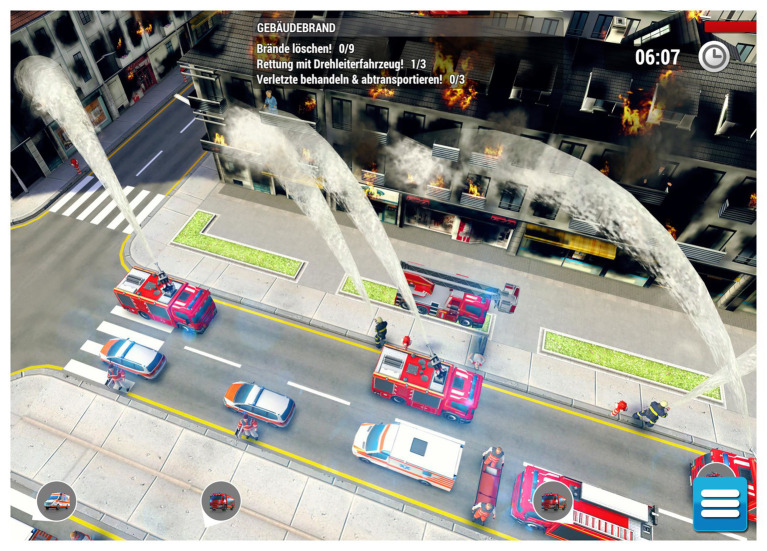
An example scene from scenario *Fire*.

The game was played from an isometric view where the viewing angle is shifted, creating a three-dimensional effect and showing some details of the environment that are not visible when viewed directly from above or from the side (see Figure 1). Participants had to choose an appropriate command from an action menu by clicking on an available emergency force, and then select a target of the requested action by clicking on the desired object. For instance, participants clicked on an emergency doctor who would then be ordered to serve a respective victim, or on a firefighter who would be ordered to put out a fire or to free a person trapped in a car. Interaction with the game was realized using a conventional computer mouse only.

After getting familiar with the game by playing an introductory tutorial and a training scenario, participants completed two target scenarios: *Fire* and *Train Crash*. Each scenario had to be played with three levels of difficulty: *easy*, *medium*, and *hard*. The difficulty levels and the scenarios differed with regard to the number of tasks to be accomplished and the number of personnel to be coordinated within a given period of time. At the beginning of each level, the number of tasks was equal for all players. Whereas, the number of victims was held constant, which means that no new victims were added during a game, the number of fires depended very much on the performance of players and therefore could grow rapidly (i.e., by fires spreading to adjacent buildings or objects if not extinguished). As the increasing task-density across levels and scenarios required not only more actions but also better coordination and prioritization, we expected that cognitive load of participants would increase with increasing difficulty of levels and scenarios. Additionally, there was a time limit for each level and scenario to impose time pressure onto participants. A summary of all parameters describing the task difficulty of each scenario and each level can be found in Table 1.

**Table 1:** Overview over the initial game parameters.

Scenario/Game parameters	Difficulty		
	Easy	Medium	Hard
***Scenario: Fire***			
Time limit (s)	450	450	450
*Tasks – total*	*8+*	*13+*	*18+*
Victims	2	3	4
Fires	4+	7+	10+
Ladder rescues	2	3	4
*Resources – total*	*9*	*12*	*15*
Doctors	1	2	2
Paramedics	1	2	2
Fire fighters	4	4	6
Fire trucks	2	3	4
Ladder trucks	1	1	1
***Scenario: Train crash***			
Time limit (s)	600	600	600
*Tasks – total*	*20+*	*30+*	*40+*
Victims	10	15	20
Cars to cut	7	10	13
Fires	3+	5+	7+
*Resources – total*	*10*	*14*	*18*
Doctors	2	3	4
Paramedics	3	5	6
Fire fighters	4	4	6
Fire trucks	1	2	2

*The number of fires depended on players’ performance and might grow. These cases are marked by the “+” sign*.

#### Training Scenario

The learning sequence involved a car accident at an intersection. The players’ task was to free all persons trapped in the crashed vehicles, treat them for health issues and transport them to the hospital. The time limit for this scenario was set to 5 min.

#### Fire

In this scenario, participants had to fight a burning building block. In addition, some residents had to be freed from the burning house, treated for health issues, and transported to the hospital. The number of fires varied depending on the players’ performance in extinguishing fires and could eventually increase rapidly. The time limit was set to 7.5 min.

#### Train Crash

This scenario depicted a train crashing into a building, causing a quick-spreading fire. The task was to free trapped passengers from the train, treat them for health issues, and then transport them to the hospital. At the same time, numerous fires had to be extinguished. In this scenario, the number of fires also varied depending on the players’ extinguishing performance. An additional difficulty was to protect emergency doctors working near a fire. The time limit for this scenario was set to 10 min.

### Measures

In the current study, cognitive load was measured by means of two methods. The objective estimation of cognitive load was performed using behavioral in-game metrics, which were defined in line with the TBRS model by Barrouillet et al. (2004). For the validation of these metrics, we acquired a subset of the NASA-TLX questionnaire as a widely accepted and thoroughly evaluated subjective instrument (i.e., *mental demand*, *time demand*, and *effort*). The details for both assessment strategies are provided below. Gaming performance was reflected by a binary indicator of whether the game was completed successfully within a given time limit or not. Additional personal information on participants such as age, gaming experience, and sex were collected prior to the experiment using a self-report questionnaire. To measure gaming experience, we asked participants to indicate how often they play (online) digital games on a 5-point Likert scale (“never,” “several times a year,” “several times a month,” “several times a week,” “every day”).

#### Behavioral In-Game Metrics

According to the TBRS Model (Barrouillet et al., 2004), working memory represents the core system of cognition dedicated to the processing and storage of information, whereby both storage and processing components are normally required for the execution of a cognitive task. In situations with pre-defined pace, cognitive load can be estimated as a relation between the time during which participants’ attention is captured by the processing of information and the total time available. This model was well evaluated on modified span tasks with a pre-defined pace (Barrouillet and Camos, 2015). In the current study, we applied this metric to a more general situation where the pace is only indirectly determined by the nature of the task and inherent time pressure.

The same task can capture attention to varying degrees in different persons, depending on their cognitive resources, which may differ, e.g., through experience or training (Case et al., 1982; Babiloni, 2019). This means that under time pressure, a person experiencing lower demands on her/his attentional resources for the task-processing component may deliberately increase her/his processing speed (task density) without affecting his/her memory component, whereas a person experiencing higher attentional demands would not be able to do so. Accordingly, when these two hypothetical persons were presented with a block comprising a certain number of tasks under time pressure (*action block*), one would observe two activity phases. In a first phase (*burst*) one would see both persons performing the presented tasks at a maximum speed. In a second phase (*idle*), they would have to wait until the end of the current *action block* until the subsequent *action block* begins. During the idle phase, both persons can only observe how their actions during the *burst* played out.

Assuming that both persons have operated at their limits, their cognitive load in the *burst* phase would be equivalent, that is, at maximum. In contrast, the duration of the *burst* phase would be different. Therefore, the cognitive load of the entire *action block* could be estimated by the relation of the duration of the *burst* phase to the total duration of the *action block* (see Equation 1). In terms of the TBRS model, this implies that the person experiencing lower demands has more temporal processing resources left and might therefore also be able to solve more difficult tasks whereas the other person has lesser resources left for time-based sharing.

$$temporalaction\;density\;decay\;\left(TADD\right)=\frac{burst}{burst+idle}\;\left(Equation\;1\right)$$

We transferred these assumptions to the situation of the game or gameplay, respectively. As a result, the following three in-game metrics were derived.

##### Normalized Gaming Time

The most obvious, but also the most basic option is to work with time-limited levels and to consider the entire level as an *action block*, while the *burst* phase would correspond to the factual gaming time and the *idle* phase to the time remaining until the end of the level. Based on this consideration, the total cognitive load for the entire level could be estimated. As this metric equals one for persons who failed at a game level and has a potential range between 0 and 1 for those who complete the respective level, it directly represents success in the game or level, respectively. Therefore, it can be seen as a performance in-game metric, which however can only be calculated retrospectively once the level has been completed.

##### TADD

A more fine-grained option would be to take a closer look at the course of the game action and to try identifying smaller *action blocks* within each level. This can be done by means of the following rationale: In the game, participants have to coordinate a set of tasks to be accomplished by a set of emergency personnel by prioritizing tasks and resources as quickly as possible (*burst* phase). When no more resources are available (i.e., when all emergency personnel are distributed to existing tasks and busy), an inevitable break occurs (*idle* phase). This *idle* phase lasts until the first emergency personnel are ready to take up a new task (beginning of the *burst* phase of the new *action block*). This theoretical approach can be applied to a range of different learning scenarios that can be found in (game-based) simulations where tasks have to be prioritized and teams/resources to be managed, e.g., utilizing elements of (real-time) strategy games for training managerial skills (Simons et al., 2020), computer programming (Muratet et al., 2009), or mathematics problem solving (Hernández-Sabaté et al., 2016).

###### Initial TADD

For predictive (and adaptive) purposes, it would be ideal to base cognitive-load estimations on very early *action blocks* within each level of a game. Therefore, we defined the *TADD* calculated for the very first *action block* of each game level as the *initial TADD*. The initial TADD comprises the time from the first user action until the first assigned emergency personnel becomes free again. The advantage of this measure is that it can be calculated during the first minutes of the gameplay and thus be used for near-real-time predictions and adaptations.

###### Mean TADD

In addition to the *initial TADD*, we also calculated a *mean TADD*, reflecting the average of *TADD* for all identified *action blocks* per level. This metric can, of course, also be calculated only retrospectively and was used mainly for an additional validation of *initial TADD*.

#### NASA-TLX

The NASA-TLX (Hart and Staveland, 1988) is a multidimensional instrument for the assessment of subjective workload, with good psychometric properties and a very high degree of acceptance in the research community (Hart, 2006). It consists of six items, estimating different aspects of subjective workload from 0 to 100 points with steps of five points, resulting in a 21-level scale. The dimensions of the NASA-TLX correspond to various theories that distinguish between physical, mental, and emotional demands imposed on an operator (Hart, 2006). For the current study, we relied on a subset of these items to specifically assess the mental facet of workload, i.e., *mental demand*, *temporal demand*, and *effort*. Using various subsets of items is quite common when investigating specific facets of workload (Temple et al., 1997; Haerle et al., 2013). Moreover, focusing on specific items allows for a time-efficient assessment of participants’ workload, which was particularly important for the current study as subjective workload was assessed after each level of difficulty for each scenario to be able to associate behavioral and subjective indices of cognitive load for the different scenarios and difficulty levels.

### Experiment Procedure

**Figure 2:**
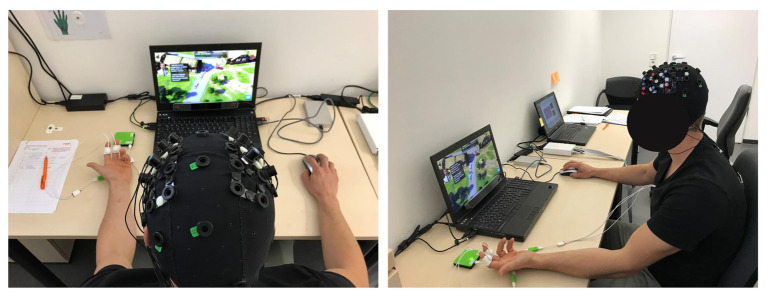
Experimental setup.

The study took place in a quiet laboratory under constant light conditions. The serious game was presented on a notebook with 16″ screen providing a 1,920 × 1,080 resolution (see Figure 2). All instructions were presented in German. The study was implemented in a within-subject design, which means that each participant completed all scenarios and levels. The game started with an introductory tutorial and a training scenario directly after welcoming participants and collecting demographic data. Each game level was followed by a brief assessment of subjective cognitive state through an adapted NASA-TLX survey.

## Results

### Statistical Analyses

For statistical analyses, we used R (R Core Team, 2020) with the *lme4* package (Bates et al., 2014) to perform generalized linear mixed-effects analyses as well as the *multcomp*, *emmeans* packages (Hothorn et al., 2008; Lenth et al., 2019) to conduct the *post hoc* comparisons described below. The *p*-values were obtained by likelihood ratio tests of the full model with the effect in question tested against a reduced model without the effect in question and were specified in further model analyses. Tukeys’ adjustment method was used for multiple comparisons. We used the *report* package (Makowski and Lüdecke, 2019) to support the description of the results. Standardized parameters were obtained by fitting a model on a standardized version of the dataset. Effect sizes were labeled following the recommendations by Funder and Ozer (2019) and (Chen et al., 2010) for linear and generalized linear models, respectively. No obvious deviations from homoscedasticity or normality were revealed using visual inspection of residual plots.

The final composition of tested models was determined by pairwise likelihood ratio tests. Thereby, the null model, which only contained test subjects as a random factor, was stepwise extended by fixed effects for scenario, difficulty, gaming experience, age, and gender. According to this procedure consideration of gaming experience, age and gender did not improve model fit significantly beyond the model only incorporating fixed effects of scenario and difficulty. For this reason gaming experience, age and gender were not considered in further analyses. In all models, we considered the effect of the two scenarios as random.^1^ because we were primarily interested in relations between in-game metrics, subjective ratings, and difficulty levels within scenarios, regardless of the gaming scenario (for an overview of the composition and main outcomes of mixed-effect analyses see Table 2).

**Table 2:** Overview of the mixed model analyses performed.

	Outcome	Effects		*p*
		Fixed	Random intercepts	
***Manipulation check***				
NASA-TLX	Mental demand	Difficulty	Participant, scenario	<0.001
	Time demand	Difficulty	Participant, scenario	<0.001
	Effort	Difficulty	Participant, scenario	<0.001
Performance	Failure/success	Difficulty	Participant, scenario	<0.001
***In-game metrics vs. subjective cognitive workload (NASA-TLX)***				
Normalized gaming time (NGT)	Mental demand	NGT	Participant, scenario	<0.001
	Time demand	NGT, scenario	Participant	<0.001
	Effort	Normed GT	Participant, scenario	<0.001
Initial TADD	Mental demand	Initial TADD	Participant, scenario	<0.001
	Time demand	Initial TADD	Participant, scenario	<0.001
	Effort	Initial TADD	Participant, scenario	<0.001
Mean TADD	Mental demand	Mean TADD	Participant, scenario	0.001
	Time demand	Mean TADD	Participant, scenario	0.003
	Effort	Mean TADD	Participant, scenario	<0.001
***In-game metrics vs. gaming performance***				
	Failure/success	Initial TADD	Participant, scenario	<0.001
	Failure/success	Mean TADD, scenario	Participant	<0.001

*Gaming performance was represented by the binary indicator of whether the game was completed successfully, i.e., all fires extinguish and all injured persons transported to the hospital (success) or not (failure). The p-values were obtained by likelihood ratio tests of the full model with the fixed effect in question against the reduced model without the fixed effect in question*.

For an overview of correlations among variables assessed in the current study, please see Table 3. Prediction of game performance was conducted using the Python (Van Rossum and Drake, 2009) module *scikit-learn* (Pedregosa et al., 2011). In particular, we used linear discriminant analysis with Leave-One-Subject-Out-Cross-Validation to train and test the models and permutation tests for model comparisons.

**Table 3:** Correlations matrix of variables considered in the present study.

	2	3	4	5	6	7	8	*9*	*10*	*11*
1. Difficulty	−0.636^**^	0.265^**^	0.529^**^	0.397^**^	0.000	0.000	0.000	0.804^**^	0.455^**^	0.479^**^
2. Gaming success	*–*	−0.322^**^	−0.590^**^	−0.465^**^	−0.226^**^	−0.147	0.142	−0.807^**^	−0.342^**^	−0.439^**^
***NASA-TLX***										
3. Mental demand		*–*	0.747^**^	0.900^**^	0.190^*^	0.122	−0.004	0.343^**^	0.139	0.185^*^
4. Time demand			*–*	0.835^**^	0.128	0.087	−0.026	0.650^**^	0.254^**^	0.402^**^
5. Effort				*–*	0.214^*^	0.147	−0.035	0.482^**^	0.201^*^	0.269^**^
***Covariates***										
6. Age					*–*	−0.053	−0.233^**^	0.176^*^	0.210^*^	0.171^*^
7. Sex						*–*	−0.182^*^	0.141	−0.055	−0.050
8. Gaming expertize							*–*	−0.129	−0.097	−0.135
***In-game metrics***										
9. NGT								*–*	0.460^**^	0.507^**^
10. Mean TADD									*–*	0.606^**^
11. Initial TADD										*–*

* Correlation is significant at the 0.05 level;

** Correlation is significant at the 0.01 level.

*Pearson 2-tailed correlations.* *The purpose of this summary is to give a first very general impression of the relations between the parameters, as presented values neither has been corrected for multiple comparisons, nor have repeated measurements been taken into account*.

### Manipulation Check

#### Subjective Ratings

To test whether the experimentally induced levels of task difficulty of the game are reflected in subjective workload measurements we ran linear mixed-effect models with the fixed factor difficulty and random intercepts for participants and scenarios on the relationship between selected items of NASA-TLX (*mental demand, time demand, effort*) and levels of difficulty (*easy, medium, hard*).

##### Mental Demand – Difficulty

**Figure 3:**
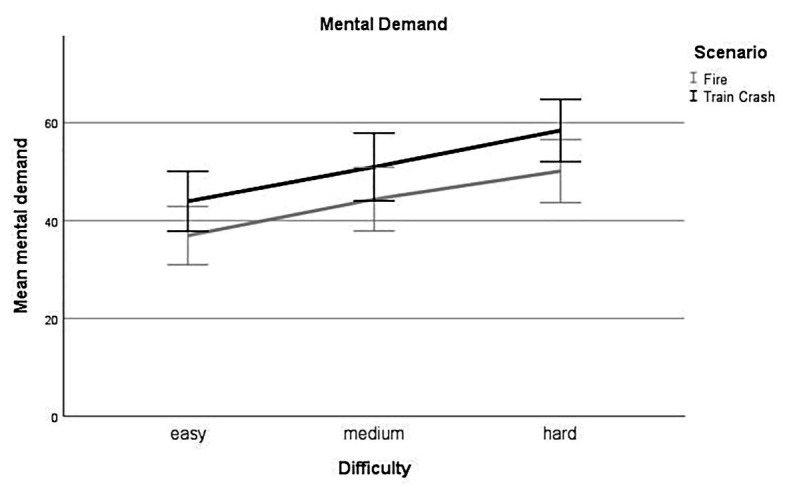
Mental demand. Mean perceived mental demand for all levels of difficulty (*easy*, *medium*, *hard*) for each scenario (*Fire*, *Train Crash*). Error bars depict ±2 *SE*, which corresponds to 95% *CI*.

Linear mixed-effect analysis revealed a significant main effect of difficulty [*χ*^2^(2) = 79.87, *p* < 0.001] on the subjective rating of mental demand. The models’ total explanatory power was substantial (conditional *R^2^* = 0.82, marginal *R^2^* = 0.06). Within this model perceived *mental demand* was significantly higher for *medium* difficulty levels compared to *low* difficulty levels, this effect can be considered as small (*beta* = 7.23, *SE* = 1.42, *std. beta* = 0.32, *p* < 0.001; see Figure 3); also, perceived *mental demand* was significantly higher for *high* difficulty levels compared to *low* difficulty levels. This effect can be considered as medium (*beta* = 13.83, *SE* = 1.42, *std. beta* = 0.61, *p* < 0.001; see Figure 3). *Post hoc* comparisons showed significant differences for all combinations of difficulty levels. Participants rated their mental demand higher during levels with higher experimentally induced task difficulty.

##### Time Demand – Difficulty

**Figure 4:**
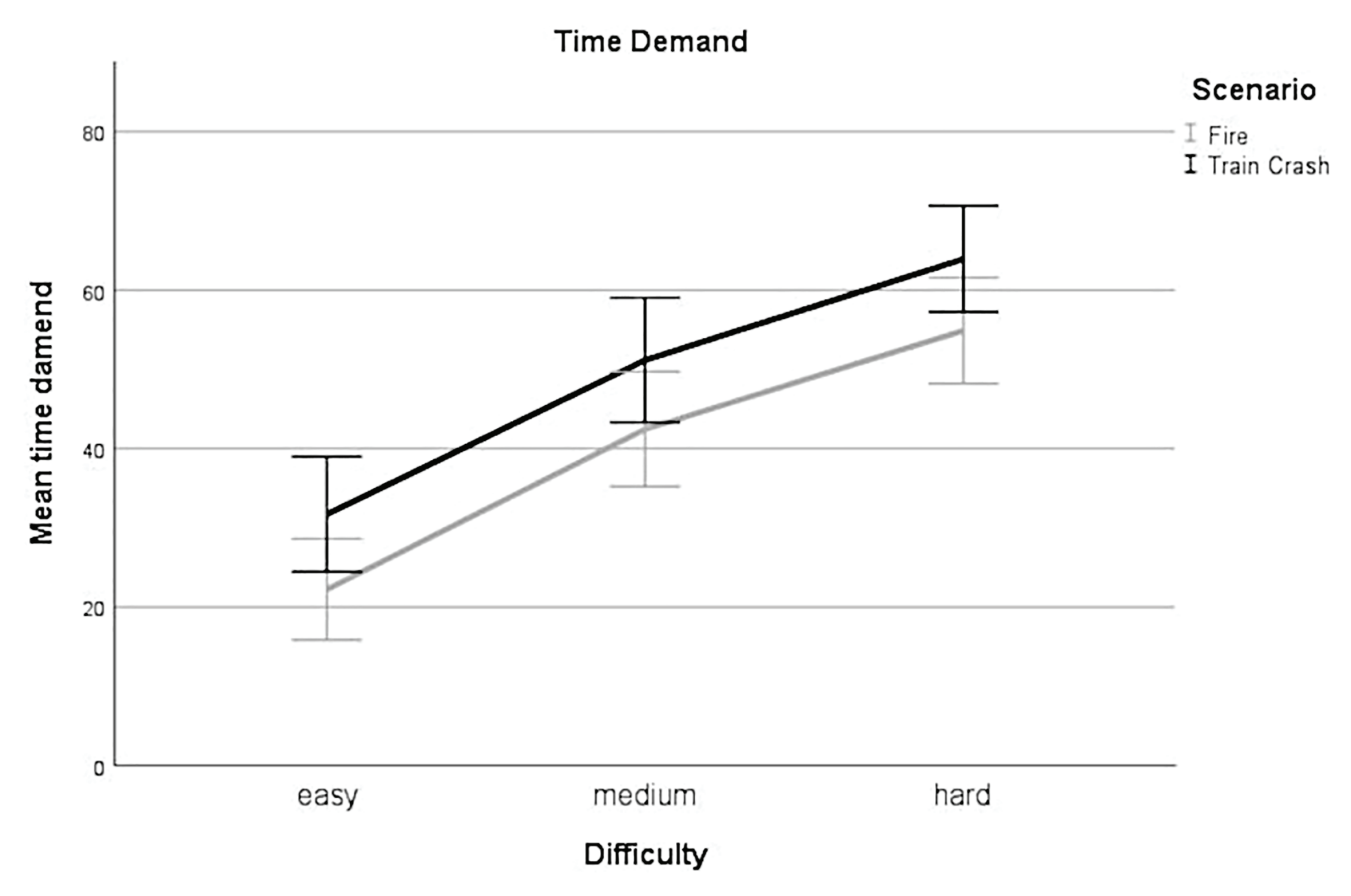
Time demand. Mean perceived time demand for all levels of difficulty (*easy*, *medium*, *hard*) for each scenario (*Fire*, *Train Crash*). Error bars depict ±2 *SE*, which corresponds to 95% *CI*.

Linear mixed-effect analysis identified a significant main effect of difficulty on the subjective rating of time demand [*χ*^2^(2) = 140.48, *p* < 0.001]. The models’ total explanatory power was substantial (conditional *R^2^* = 0.66, marginal *R^2^* = 0.23). Perceived *time demand* was significantly higher for levels with *medium* difficulty compared to levels with *low* difficulty, this effect can be considered large (*beta* = 19.84, *SE* = 2.36, *std. beta* = 0.71, *p* < 0.001; see Figure 4); perceived *time demand* was significantly higher for *hard* difficulty levels compared to *low* difficulty levels, this effect can be considered very large (*beta* = 32.45, *SE* = 2.36, *std. beta* = 1.17, *p* < 0.001; see Figure 4). *Post hoc* comparisons showed significant differences for all combinations of difficulty levels. Participants rated their time demand higher during levels with higher experimentally induced task difficulty.

##### Effort – Difficulty

**Figure 5:**
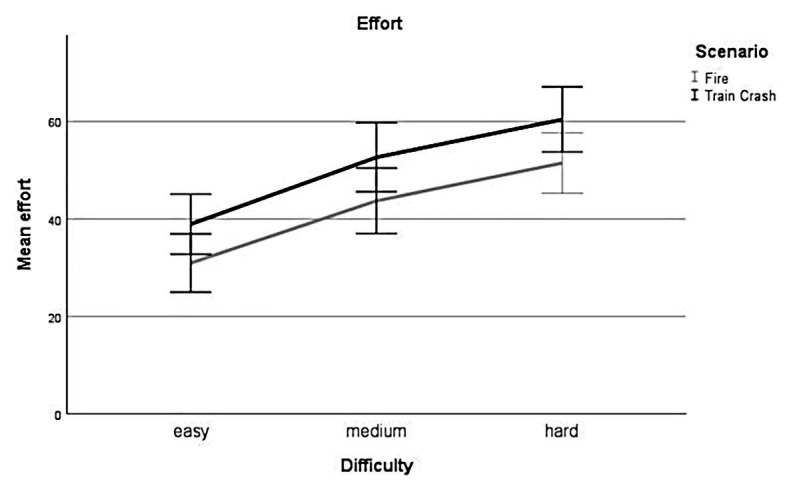
Effort. Mean perceived effort for all levels of difficulty (*easy*, *medium*, *hard*) for each scenario (*Fire*, *Train Crash*). Error bars depict ±2 *SE*, which corresponds to 95% *CI*.

Linear mixed-effect analysis showed a significant main effect of difficulty on the subjective rating of effort [*χ*^2^(2) = 105.97, *p* < 0.001]. The models’ total explanatory power was substantial (conditional *R^2^* = 0.73, marginal *R^2^* = 0.13). Within this model perceived mental *effort* was higher for *medium* difficulty levels compared to *low* difficulty levels, this effect can be considered as medium and significant (*beta* = 13.24, *SE* = 1.84, *std. beta* = 0.55, *p* < 0.001; see Figure 5), whereas perceived mental *effort* was higher for *hard* difficulty levels compared to *low* difficulty levels, this effect can be considered as very large and significant (*beta* = 21.01, *SE* = 1.84, *std. beta* = 0.87, *p* < 0.001; see Figure 5). *Post hoc* comparisons showed significant differences for all combinations of difficulty levels. Participants rated their effort higher during levels with higher experimentally induced task difficulty.

#### Performance – Difficulty

**Figure 6:**
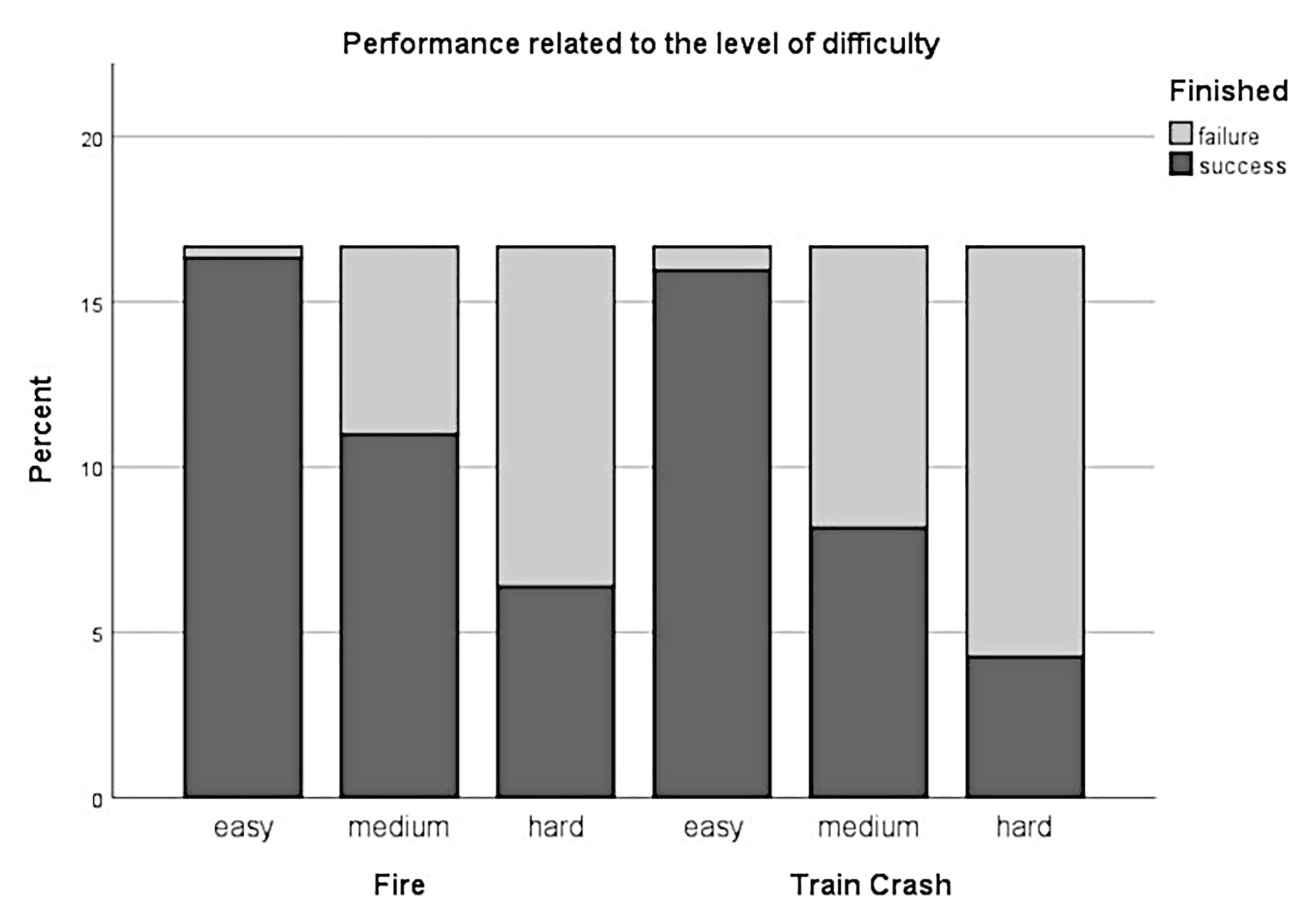
Performance in relation to the level of difficulty. Stacked histogram showing the percentage of successes/failures (i.e., whether the participants were able to rescue all victims and extinguish all fires within a defined time limit) for all levels of difficulty (*easy*, *medium*, *hard*) over both scenarios (*Fire* and *Train Crash*).

To evaluate, whether the levels of task difficulty are also reflected in gaming performance we fitted a logistic mixed-effect model on the relationship between the binary indicator of whether the game was completed successfully or not and the three difficulty levels. As we were primarily interested in the effect of difficulty levels, we considered difficulty as a fixed effect and added random intercepts for participants and scenarios. The generalized linear mixed-effect analysis revealed a significant main effect of difficulty [*χ*^2^(2) = 115.39, *p* < 0.001]. The models’ total explanatory power was substantial (conditional *R^2^* = 0.67, marginal *R^2^* = 0.49). Within this model we found that gaming performance was poorer for *medium* difficulty levels compared to *low* difficulty levels, this effect can be considered as large and significant (*beta* = −3.77, *SE* = 0.72, *std. beta* = −3.77, *p* < 0.001; see Figure 6); gaming performance was poorer for *hard* difficulty levels compared to *low* difficulty levels, this effect can be considered as large and significant (*beta* = −5.22, *SE* = 0.78, *std. beta* = −5.2, *p* < 0.001; see Figure 6). *Post hoc* comparisons also showed significant differences for all combinations of difficulty levels. Participants performed more poorly during levels with higher experimentally induced task difficulty.

### Subjective Ratings vs. In-Game Metrics

To verify whether the calculated in-game metrics is able to predict the subjectively experienced cognitive load of participants we ran a linear mixed-effect model separately for each metric and the NASA-TLX item. As we were primarily interested in the relation between the in-game metrics and the subjective ratings regardless of the gaming scenario, we defined in-game metrics as fixed factors and added random intercepts for participants and scenarios.

#### Mental Demand – Normalized Gaming Time

Linear mixed-effect analysis indicated a significant effect of *normalized gaming time* [NGT; *χ*^2^(1) = 104.33, *p* < 0.001] on self-reported mental demand. The models’ total explanatory power was substantial (conditional *R^2^* = 0.83, marginal *R^2^* = 0.10). Within this model, higher *NGTs*, i.e., participants who took longer to finish the level or even failed, was associated significantly with higher perceived mental demand, this effect can be considered as small (*beta* = 38.73, *SE* = 3.38, *std. beta* = 0.31, *p* < 0.001).

#### Time Demand – Normalized Gaming Time

Linear mixed-effect analysis revealed a significant effect of *NGT* [*χ*^2^(1) = 141.08, *p* < 0.001] on self-reported time demand. The models’ total explanatory power was substantial (conditional *R^2^* = 0.72, marginal *R^2^* = 0.38). Within this model, we found that higher *NGTs*, i.e., participants who took longer to finish a level or even failed, was significantly associated with higher perceived time demand, this effect can be considered as medium (*beta* = 0.91, *SE* = 5.59, *std. beta* = 0.60, *p* < 0.001); the effect of scenario was not significant (*beta* = 1.16, *SE* = 1.80, *std. beta* = 0.04, *p* = 0.517).

#### Effort – Normalized Gaming Time

Linear mixed-effect analysis showed a significant effect of *NGT* [*χ*^2^(1) = 125.65, *p* < 0.001] on self-reported mental demand. The models’ total explanatory power was substantial (conditional *R^2^* = 0.73, marginal *R^2^* = 0.19). Within this model, we found that higher *NGT*, i.e., participants who took longer to finish the level or even failed, was significantly associated with higher perceived effort, this effect can be considered as medium (*beta* = 56.86, *SE* = 4.42, *std. beta* = 0.43, *p* < 0.001).

#### Mental Demand – Initial TADD

Linear mixed-effect analysis revealed a significant effect of *initial TADD* [*χ*^2^(1) = 13.74, *p* < 0.001] on self-reported mental demand. The models’ total explanatory power was substantial (conditional *R^2^* = 0.76, marginal *R^2^* = 0.01). Within this model we found that higher *initial TADD*, i.e., participants who took longer to allocate the available personnel to the tasks to be done to during the first *action block*, was significantly related to higher perceived mental demand, this effect can be considered as very small (*beta* = 13.71, *SE* = 3.64, *std. beta* = 0.12, *p* < 0.001).

#### Time Demand – Initial TADD

Linear mixed-effect analysis identified a significant effect of *initial TADD* [*χ*^2^(1) = 31.31, *p* < 0.001] on self-reported time demand. The models’ total explanatory power was substantial (conditional *R^2^* = 0.45, marginal *R^2^* = 0.07). Within this model we found that higher *initial TADD*, i.e., participants who took longer to allocate the available personnel to the tasks to be done to during the first *action block*, was associated with higher perceived time demand, this effect can be considered as small and significant (*beta* = 38.10, *SE* = 6.60, *std. beta* = 0.27, *p* < 0.001).

#### Effort – Initial TADD

Linear mixed-effect analysis revealed a significant effect of *initial TADD* [*χ*^2^(1) = 22.88, *p* < 0.001] on self-reported mental demand. The models’ total explanatory power was substantial (conditional *R^2^* = 0.61, marginal *R^2^* = 0.04). Within this model we found that higher *initial TADD*, i.e., participants who took longer to allocate the available personnel to the tasks to be done to during the first *action block*, was significantly associated with higher perceived effort, this effect can be considered as very small (*beta* = 23.89, *SE* = 4.88, *std. beta* = 0.30, *p* < 0.001).

#### Mental Demand – Mean TADD

Linear mixed-effect analysis showed a significant effect of *mean TADD* [*χ*^2^(1) = 11.93, *p* < 0.001] on self-reported mental demand. The models’ total explanatory power was substantial (conditional *R^2^* = 0.76, marginal *R^2^* = 0.01). Within this model higher *mean TADD*, i.e., participants who in average took longer to allocate the available personnel to the tasks to be done to during all defined *action blocks*, was significantly associated with higher perceived effort, was significantly linked to higher perceived *mental demand*, this effect can be considered as very small (*beta* = 30.62, *SE* = 8.73, *std. beta* = 0.11, *p* < 0.001).

#### Time Demand – Mean TADD

Linear mixed-effect analysis revealed a significant effect of *mean TADD* [*χ*^2^(1) = 8.83, *p* = 0.003] on self-reported time demand. The models’ total explanatory power was substantial (conditional *R^2^* = 0.40, marginal *R^2^* = 0.02). Within this model higher *mean TADD*, i.e., participants who in average took longer to allocate the available personnel to the tasks to be done to during all defined *action blocks*, was associated with higher perceived time demand, this effect can be considered as very small and significant (*beta* = 49.37, *SE* = 16.38, *std. beta* = 0.15, *p* < 0.01).

#### Effort – Mean TADD

Linear mixed-effect analysis identified a significant effect of *mean TADD* [*χ*^2^(1) = 13.31, *p* < 0.001] on self-reported mental demand. The models’ total explanatory power was substantial (conditional *R^2^* = 0.59, marginal *R^2^* = 0.02). Within this model higher *mean TADD*, i.e., participants who in average took longer to allocate the available personnel to the tasks to be done to during all defined *action blocks*, was significantly related with higher perceived effort, this effect can be considered as very small (*beta* = 43.97, *SE* = 11.85, *std. beta* = 0.15, *p* < 0.001).

### Performance vs. In-Game Metrics

To verify whether the calculated in-game metrics would be able to predict the final performance of a given difficulty level, we ran a generalized linear mixed-effect model separately for the in-game metric *initial TADD* as well as for *mean TADD* and the binary indicator identifying whether the participants were able to extinguish all fires and transport all injured persons to the hospital (success) or not (failure). Since *NGT* basically was a performance measure, it predicts gaming success perfectly and cannot be used as a predictor variable in the mixed model. As we were primarily interested in the relation between in-game metrics and performance regardless of gaming scenario, we defined in-game metrics as fixed factors and added random intercepts for participants and scenarios.

#### Performance – Initial TADD

**Figure 7:**
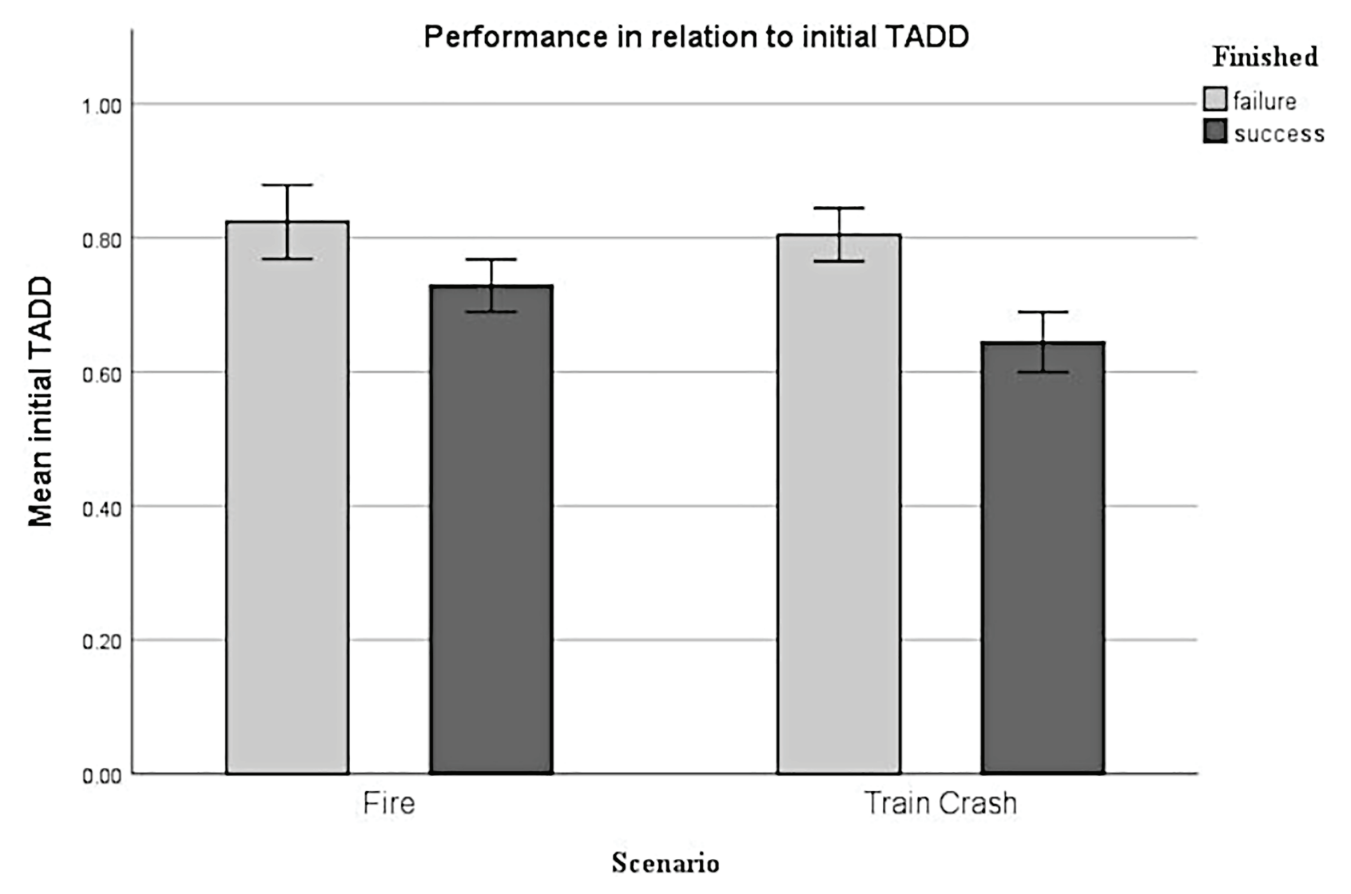
Performance in relation to initial TADD. Error bars depict +/- 2 SE, which corresponds to 95% CI.

Generalized linear mixed-effect analysis revealed a significant effect of *initial TADD* [*χ*^2^(1) = 28.96, *p* < 0.001] on performance. The models’ total explanatory power was moderate (conditional *R^2^* = 0.22, marginal *R^2^* = of 0.14). Within this model we found that higher *initial TADD* was significantly linked to lower performance, this effect can be considered as small (*beta* = −3.86, *SE* = 0.79, *std. beta* = −0.76, *p* < 0.001; see Figure 7).

#### Performance – Mean TADD

Generalized linear mixed-effect analysis indicated a significant effect of mean TADD on performance [*χ*^2^(1) = 10.21, *p* < 0.001]. The models’ total explanatory power was weak (conditional *R^2^* = 0.13, marginal *R^2^* = 0.07). Within this model the higher initial TADD was significantly associated with lower performance, this effect can be considered as very small (beta = −5.54, SE = 1.85, std. beta = −0.47, *p* < 0.01), whereas the effect of scenario was not significant (beta = −0.34, SE = 0.26, std. beta = −0.34, *p* < 0.190).

### Performance Prediction

To verify whether the *initial TADD* may be suitable for real-time or near-real-time prediction of performance (i.e., finished level successfully vs. failed) of the given level we used linear discriminant analyses with Leave-One-Subject-Out Cross-Validation. These demonstrated a 67.38% accuracy in scenario *Fire* and a 64.53% in the *Train Crash* scenario. However, permutation tests comparing the models’ performance with the performance of models predicting randomly permuted outcomes showed that only for the *Train Crash* scenario this was significantly above the score of random models (*Fire*: random models mean accuracy: 67.28%, *p* = 0.886; *Train Crash*: random models mean accuracy: 55.37%, *p* < 0.001).

A linear discrimination analysis using the three NASA-TLX subscales in the performance scenario showed an average accuracy of 73.04%. A permutation test showed that this accuracy was significantly higher than models with randomly permuted outcomes (random models mean accuracy: 53.81%, *p* < 0.001). However, a permutation test showed that the accuracy of this model was not significantly different from the model using only our in-game metric (*p* = 0.09).

## Discussion

The current study aimed at evaluating a practicable, parsimonious, and reliable approach for the online assessment of cognitive load in serious games, which is suitable for cognitive load prediction during realistic gaming setups with a similar structure to the game used in the study, i.e., a (real-time) strategy like serious game. Based on the TBRS model of Barrouillet et al. (2004) we defined several in-game metrics (*initial TADD*, *mean TADD*, *NGT*) for describing the behavior and performance in an emergency simulation game. The results indicated that it seems indeed possible to use these simple in-game metrics to reliably assess and predict cognitive load based on a theory-driven approach. In the following, we critically discuss these results in greater detail.

First of all, we aimed at verifying whether the experimentally induced difficulty levels of the serious game were actually able to induce substantial differences in cognitive load for the participants. This manipulation check was an important prerequisite for examining our main scientific hypotheses. Results clearly indicated that increased difficulty (e.g., in terms of more personnel to coordinate and more tasks to execute under a constant time limit) indeed resulted in significantly higher subjective ratings of cognitive load accompanied by significantly poorer performance. This substantiated our expectations and indicated that the intended manipulation of cognitive load by means of game-difficulty levels worked as intended.

Further analyses showed that all three proposed in-game metrics can be considered valid to significantly predict self-reported workload as well as actual gaming success. In particular, *NGT* (i.e., ratio of actual playing time to total time available per game level) showed a more pronounced effect on subjective workload ratings as compared to *initial* and *mean TADD* (*temporal action density decay*; i.e., ratio of active playing time *burst* to total time available in the first *action block* and averaged over all *action blocks* defined per level). This may be due to the fact that the former measure was directly related to performance and thus most sensitive to subjectively experienced cognitive load. For instance, participants were able to develop a feeling for how well they have performed the game at the time of the survey and thus experienced failure may have resulted in higher perceived cognitive load as compared to known success (Hancock, 1989).

More interestingly, *initial TADD* showed a better predictive power as compared to *mean TADD* not only regarding subjective ratings of cognitive load but also in terms of the resulting performance. This suggests that early stages of gameplay may be more informative and thus more predictive for later gameplay outcomes than an aggregated score accumulated over a longer period of time. In this context, averaging *TADD* across the entire duration of the level seems to lead to a substantial loss of information for this metric.

A closer look at the construction of the game may help to better understand this difference. At the beginning of each level, a new emergency scenario was presented, and participants had to start assigning tasks to the available emergency personnel soon. That is, right at the beginning of the level participants had to orientate themselves in a completely new situation, to plan their rescue strategy, and to implement this strategy as quickly as possible. In addition, almost the entire rescue team had to be assigned to their tasks at this point, meaning that the first action block may have been significantly longer than all subsequent blocks, which were not as clearly defined due to more constant interactions with the game.

One possible explanation for the superiority of the *initial* over the *mean TADD* might be that as the game progressed, successful players realized that they were well in time and therefore experienced less time pressure. This might have resulted in longer *burst* and shorter *idle* phases, as they were not longer operating at their maximal speed, resulting in increased *TADD* ratios in the later stages of the level. Otherwise, it also seems conceivable that the initial orientation itself plays a crucial role in the outcome of the level. As better planning in early stages of a particular task was observed to be associated with better performance in various tasks (Capon et al., 1994; Saddler et al., 2004; Wang and Gibson, 2010), *initial TADD* might also reflect more efficient planning to underlie decreased cognitive load during the initial *action block*. However, these assumptions need to be investigated in future studies.

The final aim of this study was to evaluate whether it would be possible to use in-game metrics for a real-time or near-real-time assessment of cognitive load and – based on this – a substantial performance prediction. The in-game metrics *NGT*, as well as *mean TADD*, represent summary measures, which can only be calculated retrospectively once a level has been completed. Thus, they cannot be used for predictive purposes. In contrast, *initial TADD*, which was calculated during the first minutes of gameplay, significantly predicted gaming success – at least in the *Train Crash* scenario. Moreover, it could be shown that the prediction accuracy of a model using only this metric did not significantly differ from the model using selected NASA-TLX subscales as predictors for gaming performance. Interestingly, no significant prediction could be obtained for the less difficult scenario *Fire*. Importantly, however, this may have been influenced by a crucial data issue as far more participants succeeded in the scenario *Fire* than failed, whereas in the scenario *Train Crash* this relation was more balanced. Accordingly, the difference between the two scenarios may indicate an existing floor effect for the easy scenario, indicating that the use of this metric may be suitable only for situations eliciting phases of maximum cognitive load. Whether this assumption is correct must be investigated in future studies.

In summary, the results of the current study indicated that gaming performance can be significantly predicted using *initial TADD* calculated from a short time interval at the very beginning of a new game level. This means that we were able to predict well above chance level whether the respective level would be completed successfully based on data acquired through the first tenth of the total gaming time. It is noteworthy that the quality of this prediction did not significantly differ from the prediction based on participants’ retrospective and subjective ratings using the NASA-TLX that are informed by their experienced success of failure during game play. Hence, *initial TADD* seems to qualify well for a near-real-time adaptation of game flow, not requiring considerable computing power as it is the case for more data-driven approaches (e.g., neuronal networks or deep learning based on physiological data).

### Methodological Strengths and Constraints

There are different analytical approaches to serious games (for review see: Zohaib, 2018), which are often based on data-driven probabilistic performance evaluations (Magerko et al., 2006; Spronck et al., 2006; Zook and Riedl, 2012). Simple performance data, however, often seemed insufficient for estimating cognitive and emotional states of users, such as attention, cognitive load, or emotional responses. Therefore, these cognitive states are often assessed using (neuro-)physiological data (for reviews see: Kivikangas et al., 2011), which are, however, relatively complex and laborious to acquire and computationally intensive to evaluate and are thus not always suitable for real-world applications outside the laboratory. Importantly, though, the current study demonstrated that assessment/prediction of cognitive load using simple in-game metrics is feasible. We think that there are two crucial constraints for this approach to be successful: First, a theoretically informed top-down development and second the application within an appropriate test environment.

As regards the former, we are confident that a theoretical top-down approach may be key to find parsimonious, but still reliable and generalizable solutions. Therefore, a suitable theoretical framework should be chosen in the first place. In our case, the TBRS model (Barrouillet et al., 2004) specifically emphasizes the role of time pressure as the origin of cognitive load, therefore seeming to be particularly useful for predicting workload in time-critical situations such as serious game scenarios similar to the current one, i.e., (real-time) strategy games and simulations.

With respect to the latter, the development of an appropriate testing environment is essential. As, for instance, the TBRS model was originally evaluated on very specific tasks with strong time pressure induced through pre-defined pace, we evaluated whether its predictions may generalize to more realistic applied situations. In this way, we derived two critical aspects of a test situation to make these predictions work: time pressure and time-limited blocks of tasks. By considering these aspects, we designed a gaming environment that allowed for testing the proposed metrics.

### Limitations and Open Questions

The methodological strengths and constraints of our study, however, can also be considered as limitations because it may not be possible to generalize the proposed metrics to all possible gaming situations. Presumably, they may well be used in settings with inherent time-limits and time pressure, where participants are exposed to new situations and have to manage various tasks and resources as it is the case in real-time strategy games. Other examples with similar task structures may comprise complex surgery tasks, assembly lines or time-critical emergency situations in the context of control tasks. Further testing will be required in the future to substantiate the predictive power of proposed in-game metrics in this type of situation and, possibly, to adapt the computation of these metrics appropriately.

Therefore, the current study suggests a promising perspective, but at the same time raises several questions to be explored in the future. For instance, it is not clear whether and how the predictive power of the proposed metrics is related to the given time pressure and whether they can, therefore, be used in scenarios that are less time-critical. On the other hand, it is possible that collected in-game metrics might be affected by factors other than cognitive load, such as motor processes related to the experience of the player with provided game controls, for instance. Since we used a conventional computer mouse as the only game control, we are confident that all participants were used to it and therefore the results obtained are valid in this respect. However, such general physiological processes should be taken into account and evaluated before proposed in-game metrics are generalized to different contexts. Furthermore, it should be evaluated more thoroughly why the *initial TADD* showed better performance as compared to the *mean TADD*. It might be possible, for instance, that the predictive value of the *mean TADD* (or the mean of the first few TADDs) can be improved by using other gaming situations or by sharpening the definition of *action blocks*.

### Implications and Future Perspectives

The use of simple in-game metrics for measuring cognitive load and thus deriving performance prediction yields several advantages. First, our results suggest that psychological constructs, which have traditionally been assessed explicitly using either paper-pencil or computerized questionnaires, may well be estimated more implicitly using in-game metrics, that is without causing interruption to the task at hand (cf. stealth assessment: Shute and Kim, 2014). Second, whereas the use of more complex psychophysiological measures would come with additional computational and procurement costs, systems that operate on simple in-game metrics may be made more easily accessible to the general public. More complex systems relying on resources such as neural networks are computationally rather expensive and might require substantial computing power. In contrast, simpler models for cognitive load estimation such as the one used in the current study may be easily run in parallel to the actual game on any PC without significant consumption of computing resources. Third, also complex multimodal measurement systems, which operate with sophisticated algorithms and integrate data from physiological and behavioral sources in research laboratories, may benefit from the development of simpler in-game metrics as these may be added to these more complex algorithms quite easily, thereby leading to improved classification accuracy in the future. Finally, the substitution of more complex probabilistic algorithms through simpler but reliable metrics (whenever possible) might lead to simplifications of complex models, while at the same time expanding their availability and usage. However, we feel that this may only be achieved when substantial evidence for relevant in-game metrics is based on theory rather than data alone.

### Conclusion

The present study indicated that parsimonious, but theoretically well-founded in-game metrics can be used to estimate users’ current cognitive load and, based on this, predict future gaming performance within the first tenth of the total gaming time. We applied our approach to a serious game simulating a time-critical emergency situation and requiring the management of emergency personnel. The game included different scenarios with three levels of difficulty each inducing corresponding levels of cognitive load. Based on proposed in-game metrics we were able to predict whether the respective level would be completed successfully or not well above chance level. Interestingly, the quality of this prediction did not differ significantly from a prediction based on participants’ retrospective and subjective ratings using the NASA-TLX questionnaire. To achieve this we used a rather simple model that interprets behavioral data in the light of the TBRS theoretical approach (Barrouillet et al., 2004). Based on its parsimony and the corresponding low computational power required, this model can be easily incorporated into games to create an adaptive system. Further, the measure and models introduced in this study could be used in conjunction with other adaptive features to design even more comprehensive adaptive systems that can predict performance more effectively and accurately. Taken together, our results provide promising first evidence that needs to be substantiated in future research to determine whether it is suitable for more general reliable assessments of players’ cognitive load and for respective real-time adaptations of games or game-based learning environments.

## Data Availability Statement

The raw data supporting the conclusions of this article will be made available by the authors, without undue reservation.

## Ethics Statement

The studies involving human participants were reviewed and approved by Ethics Committee of Leibniz-Instituts für Wissensmedien (IWM). The patients/participants provided their written informed consent to participate in this study. Written informed consent was obtained from the individual(s) for the publication of any potentially identifiable images or data included in this article.

## Author Contributions

NS, MN, and FW conceptualized and designed the study. NS conducted the study. NS and FW conducted the statistical analyses. NS wrote the first draft of the manuscript which was edited in several rounds with MN and FW. KM and PG provided the last rounds of edits on the manuscript. All authors revised the final manuscript. All authors contributed to the article and approved the submitted version.

### Conflict of Interest

NS was employed by the company Daimler Trucks AG.

The remaining authors declare that the research was conducted in the absence of any commercial or financial relationships that could be construed as a potential conflict of interest.

## Footnotes

^1^Two models did not converge with participants and scenarios as random effects. Therefore, we decided to run models with participants as the only random effect, considering the scenario as a fixed factor instead (see Table 2).
